# The Potential of MicroRNAs as Non-Invasive Prostate Cancer Biomarkers: A Systematic Literature Review Based on a Machine Learning Approach

**DOI:** 10.3390/cancers14215418

**Published:** 2022-11-03

**Authors:** Emilia Bevacqua, Salvatore Ammirato, Erika Cione, Rosita Curcio, Vincenza Dolce, Paola Tucci

**Affiliations:** 1 Department of Pharmacy, Health and Nutritional Sciences, University of Calabria, 87036 Rende, Italy; 2 Department of Mechanical, Energy and Management Engineering, University of Calabria, 87036 Rende, Italy

**Keywords:** tumor, microRNA, biomarker, prognosis, text mining, digitalization

## Abstract

**Simple Summary:**

Prostate cancer (PCa) is the most common cancer in men worldwide. Screening and diagnosis are based on prostate-specific antigen (PSA) blood testing and digital rectal examination. Nevertheless, these methods are not specific and have a high risk of mistaken results. This has led to overtreatment and unnecessary radical therapy; thus, better prognostic tools are urgently needed. In this view, microRNAs (miRs) appear as potential non-invasive biomarkers for PCa diagnosis, prognosis, and therapy. As the scientific literature available in this field is huge and very often controversial, we identified and discussed three topics that characterize the investigated research area by combining the big data from the literature together with a novel machine learning approach. By analyzing the papers clustered into these topics we have offered a deeper understanding of the current research, which helps to contribute to the advancement of this research field.

**Abstract:**

*Background:* Prostate cancer (PCa) is the second leading cause of cancer-related deaths in men. Although the prostate-specific antigen (PSA) test is used in clinical practice for screening and/or early detection of PCa, it is not specific, thus resulting in high false-positive rates. MicroRNAs (miRs) provide an opportunity as biomarkers for diagnosis, prognosis, and recurrence of PCa. Because the size of the literature on it is increasing and often controversial, this study aims to consolidate the state-of-art of relevant published research. *Methods:* A Systematic Literature Review (SLR) approach was applied to analyze a set of 213 scientific publications through a text mining method that makes use of the Latent Dirichlet Allocation (LDA) algorithm. *Results and Conclusions:* The result of this activity, performed through the MySLR digital platform, allowed us to identify a set of three relevant topics characterizing the investigated research area. We analyzed and discussed all the papers clustered into them. We highlighted that several miRs are associated with PCa progression, and that their detection in patients’ urine seems to be the more reliable and promising non-invasive tool for PCa diagnosis. Finally, we proposed some future research directions to help future scientists advance the field further.

## 1. Introduction

Prostate cancer (PCa) is the most commonly diagnosed cancer and the second leading cause of cancer death in men in the developed world [1], with a mortality rate expected to approximately double over the next 20 years [2]. Prostate cancer can be clinically insignificant (low-risk and localized to the prostate) or significant, in this case it is a potentially metastatic and aggressive tumor, which requires early detection, and is lethal if untreated. The prostate-specific antigen (PSA) test is used as a screening biomarker of PCa, but alone it is not indicative of the disease, therefore digital rectal examination is also required. Its diagnosis is based only on histopathological analysis of prostate biopsies. Due to the widespread use of the PSA test for PCa detection, its incidence rapidly increased, even if mortality remained stable. The main reason is that, although this test is still a gold-standard, it is not a specific biomarker, and is not very helpful in distinguishing aggressive from non-aggressive diseases, thus resulting in a high number of false positives, as well as it fails to detect indolent disease. This has led to overtreatment with radical therapy, resulting in a dramatic impact on men and their quality of life. Distinguishing the aggressive and lethal tumoral form from the indolent one is, therefore, extremely relevant to limit overtreatment and improve patient outcome. This highlights the urgent need for more specific and sensitive diagnostic and prognostic tools.

MicroRNAs (MiRs) are small non-coding RNAs that modulate gene expression and play significant roles in almost all biological pathways, influencing cancer-relevant processes, such as proliferation, apoptosis, cell cycle, invasion and migration [3]. Profiling of miRs in human cancer has generated great interest, and several studies have described their critical role in PCa pathogenesis [4,5]. More interesting is their potential use as biomarkers for the early detection, diagnosis, and prognosis of cancer. Indeed, miRs are actively released by different cell types and detectable in all human bio-fluids, especially in plasma, serum, and urine, making them suitable as circulating biomarkers for PCa [6,7]

As a considerable number of papers on both miRs and PCa are available in the literature, a systematic review approach was required to carry out a deep and comprehensive investigation of the whole literature [8]. The systematic review approach is used when the number of contributions to be analyzed is huge and, as in this case, heterogeneous and often controversial. Several studies, for example, reported that upregulated expression of miR-200 family members in PCa facilitates oncogenic activity and promotes metastasis [9], despite the prevailing opinion that under-expression of the miR-200 family promotes EMT and metastasis [10,11].

Machine learning has transformed oncological research in recent years. For instance, it has been used to classify tissue samples as benign or malignant, or for the early and automatic detection of cancer by using whole slide images [12]. The technical reason of massive machine learning adoption in medicine resides in the fast progresses in classification models, which supports the adoption of these techniques in many tools such as image-based ones [13].

This study is the first of its kind in medicine, as the machine learning classification approach has been used to locate existing studies, select and evaluate quality contributions, analyze and synthesize results and, finally, report results that can highlight clear conclusions about what is known and what is not yet known [14]. According to Petticrew [15] “a systematic review is an efficient technique for hypothesis testing, for summarizing the results of existing studies, and for assessing the consistency among previous studies”. However, the approach we used is not the traditional one used in medicine, as exemplified by the Cochrane Collaboration [16], but an innovative qualitative approach useful to provide a comprehensive/integrated analysis on all articles published in prestigious scientific journals [17]. Consequently, we performed a Systematic Literature Review (SLR) approach by using the MySLR digital platform to analyze a large number of scientific publications through a text mining method, which makes use of the Latent Dirichlet Allocation (LDA) algorithm.

Therefore, the main aim of the present study was to consolidate the state-of-art of the research published over the last 15 years on the prognostic significance of miRs expression in PCa, and on their potential use as alternative non-invasive biomarkers. This has produced a systematic mapping of the insights and knowledge gaps present in existing research, thus providing useful insights that can contribute to the development of this research field and suggesting promising directions for future research. This study represents the first attempt in which a text mining approach was applied on a sample of scientific original articles in a medical setting.

## 2. Methods

We adopted a machine learning approach to deeply analyze a large number of papers present in the scientific literature to extract latent knowledge useful for the aim of this research.

We did not contrast studies conducted with the same medical protocol, as in traditional SLR in medicine. The original research protocol adopted in this study considered scientific papers that, regardless of the adopted medical protocol, somehow dealt with potential connections between miRs and PCa.

Although traditional algorithms are developed around numerical and structured data, the information generated in the scientific literature consists of documents (papers) that are generally unstructured. Consequently, the LDA algorithm was chosen to extend machine learning applications, in order to extract information from unstructured textual data, i.e., scientific journal articles [18]. The behavior that the MySLR platform reproduces by implementing the LDA algorithm, simulates as close as possible that of a “human-like intelligence”, it can process a large amount of data, read the texts, understand their content, extract the required information, and highlight hidden connections among papers. More in detail, this approach is based on the creation of a model that is able, by analyzing texts, to autonomously identify within them a set of “topics” (or themes), to identify the topic addressed by each of them and subsequently to recognize within the various papers the presence of the topics identified above.

Therefore, we carried out a SLR to offer a complete and exhaustive overview of scientific research on the potential value of miRs as non-invasive biomarkers for PCa. This was conducted according to the Preferred Reporting Items for Systematic Reviews and Meta-Analyses (PRISMA) guidelines [19]. In order to perform this activity, we adopted the MySLR digital platform [17], a semi-automated tool supporting scientists in performing SLR, which is available at https://myslr.unical.it (accessed on 20 May 2022) upon registration.

The methodological approach is based on three steps, namely: paper location and selection, paper analysis, and results presentation, according to Denyer and Tranfield [14], as discussed below.

### 2.1. Paper Location and Selection

We searched the PubMed, Scopus, and ISI Web of Knowledge online databases up to 20 May 2022, to identify relevant studies published between 2007 (year in which the first paper in the investigated field was published) and 2022. The terms associated with the keywords were: (“microRNA” OR “miRNA” OR “miR”) AND (“prostate cancer” OR “prostate carcinoma” OR “prostate tumour” OR “prostate tumor” OR “prostate neoplasm”) AND “biomarkers” AND “prognosis”. The search string was structured in such a way that the results contained papers with at least one term from each set in the title, abstract and keywords.

### 2.2. Paper Analysis

At this stage, after removing duplicates, we examined papers (*n* = 618) to identify relations and common points among them.

Studies that met the following inclusion criteria were considered eligible:

- The study was conducted on human cells, tissue or patients with PCa (not xenografts or other animal models).
- The study measured the expression of miRs in serum/plasma/urine or cells/tissues.
- The study investigated the association between prognosis outcomes and miRs expression.

Studies were excluded if:

- The study tested the prognostic role of target genes instead of the miR itself.
- The study involved other non-coding RNAs with as yet unknown functions, such as circular RNAs, long non-coding RNAs and small nucleolar RNAs.
- The clinical study lacked key information such as hazard ratio (HR), 95% confidence intervals (CI), *p* value, and survival curves.
- The study was a review, an editorial article, a meta-analysis, a letter to editors, a short communication, a conference paper, an erratum, a chapter book, a note, a personal opinion and commentary, or a retracted publication.

We independently evaluated pertinent papers by examining titles, abstracts, and full texts matching the appropriate criteria. At the end, 213 journal articles were included in the final set of eligible documents for further topic extraction analysis.

To highlight the main research topics in the context of miRs as potential biomarkers in PCa, we performed a text mining method on the final set of 213 papers. This method is based on LDA, a statistical procedure that provides each document with a distribution along a certain number of topics. The model treats documents as topics probability distribution and topics as words distributions.

In Natural Language Processing, a topic model is a statistical model whose objective is to find the abstract “topics” (or themes) contained in a set of documents. The topics are not known a priori but are independently identified by the algorithm based on the frequency and number of occurrences of the words in the various texts. By exploiting statistics of this type, the used algorithm was able to identify three main general topics (the so-called topics) related to the keywords given by the LDA procedure present in the various texts, and to correctly assign each text its respective semantic topic. This procedure provides as output:

- k sets of relevant keywords (where each set represents a topic).
- The document-term matrix, i.e., a matrix describing how much each paper is statistically related to a specific topic (namely, the topic proportion).

### 2.3. Results Presentation

The last step of the methodological approach is elucidated in the sections “Results” and “Discussion”. The aim of this step is to clearly describe and discuss the results of the LDA procedure by means of a detailed human-based review of significant papers gathered around the three topics.

## 3. Results

**Figure 1:**
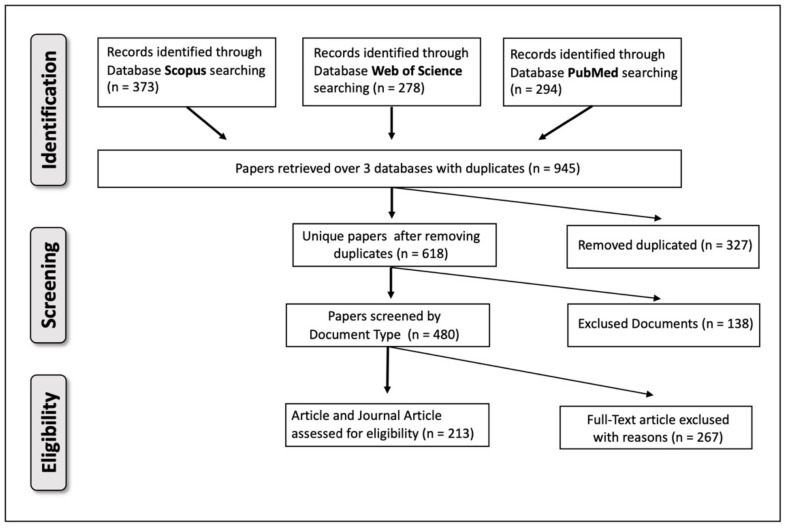
Flow diagram showing the algorithm of selection of eligible studies included in the SLR. The search process was carried out until 20 May 2022.

An overall number of 618 unique studies were retrieved from the initial literature search. Of these, 138 papers were removed as they reported non-relevant studies such as reviews, book chapters, meta-analyses, and other not relevant publications. Full-text reading and analysis resulted in removal of 267 other studies for reasons such as inability to access full text or unsatisfactory reporting of results, or that they did not meet the above inclusion criteria. Ultimately, a total number of 213 studies were considered eligible. The flowchart shown in Figure 1 elaborates the algorithm of selection of final studies for this systematic review.

**Figure 2:**
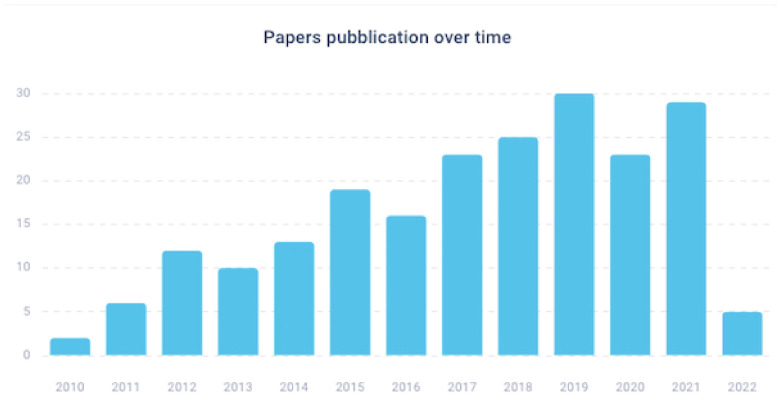
Published articles by year.

As shown in Figure 2**,** the interest of the scientific community on the issue is evident. It is not surprising that the debate around this theme has received the attention of numerous original articles over time, especially starting from 2017. In fact, if we do not consider 2022, which is still ongoing, over 60% of the articles have been published in the last 5 years.

According to the indication provided in Blei [18], we selected the k value (number of topics to be extracted) of 3, which ensured a satisfactory value of topic coherence (−1.09) [20] in unison with an easy interpretation of the results for a human reader.

**Figure 3:**
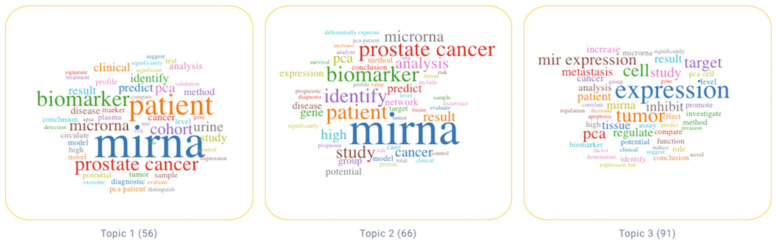
Word cloud highlighting the importance of the topic keywords.

Thanks to the LDA procedure, we identified relevant keywords associated to each of the three topics. In Figure 3, a graphical representation of the most relevant keywords for each topic is provided in the form of “word cloud”.

**Figure 4:**
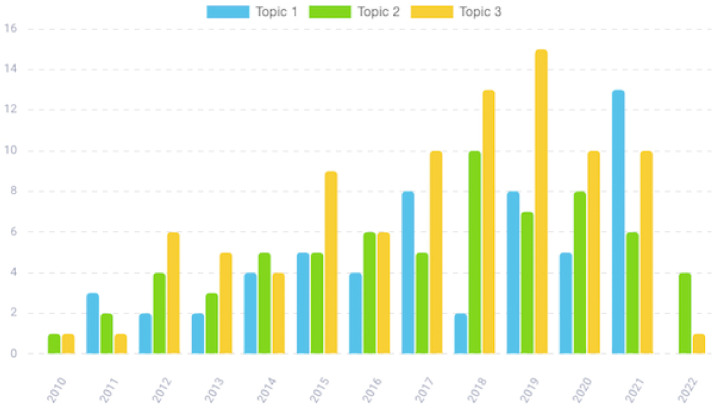
Topic papers over time.

As shown in Figure 4, although the issue under investigation has been increasingly considered by researchers over recent years, the trend of the topic papers over time is different. First, topic 1, after a fairly slow start, has had a rapid increase since 2017, and recently it has gained increasing interest (Figure 4, blue). In contrast, topic 2 has had a steady growth over time until it reached its highest peak in 2018, then its interest decreased (Figure 4, green). A similar trend can be observed for topic 3, which reached its peak of interest around 2018–2019 (Figure 4, yellow).

The three topics identified through the LDA algorithm are presented and discussed below. Then we performed a human-based review on a subset of relevant papers to infer a meaningful description of each topic. Based on the main concepts of the papers, we developed the discussion starting from topic 3, then we treated topic 2 and finally topic 1.

### 3.1. Topic 3—Study of miRs in Human PCa

**Figure 5:**
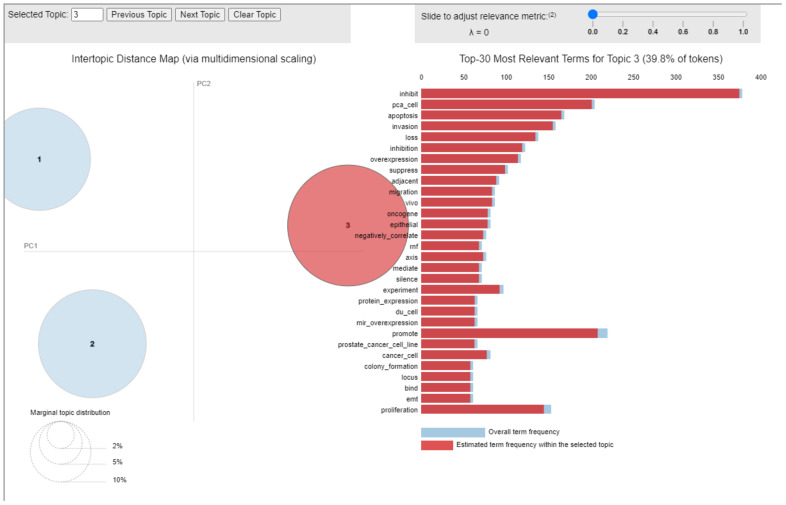
Inter-topic Distance Map related to topic 3. Circle 1 indicates topic 1, circle 2 is topic 2 and circle 3 is topic 3.

Looking at the top-30 most relevant terms and their frequency within papers grouped around the selected topic (Figure 5), and then by analyzing the 91 papers clustered into this topic, it was evident that the cornerstone of the topic was the role of miRs in PCa progression and development.

The miRs can act as oncogenes (if upregulated in PCa) or tumor suppressors (downregulated miRs) and contribute to the development and progression of tumors, thus affecting the prognosis and survival of cancer patients. Most of these papers share results obtained from in vitro experiments to explore the function of candidate miRs. Authors identified miR signatures that were able to differentiate malignant PCa from benign prostate hyperplasia. The MiR expressions were determined mostly by qPCR. Furthermore, the identification of miR target genes and their pathways played a significant role in a better knowledge of PCa. Thus, miRs can act as oncogenes or tumor suppressors in PCa by influencing multiple cancer-related processes, among which the main are cell growth and proliferation, apoptosis, migration, invasion, and metastasis, as well as epithelial to mesenchymal transition (EMT). The most relevant and representative papers clustered in the topic are summarized in Table 1, from which deregulated miRs in PCa, their putative targets and main regulatory effects on tumors, together with other pathological data, can be found.

### 3.2. Topic 2—Potential of miRs as Biomarkers in Translational Research of PCa

**Figure 6:**
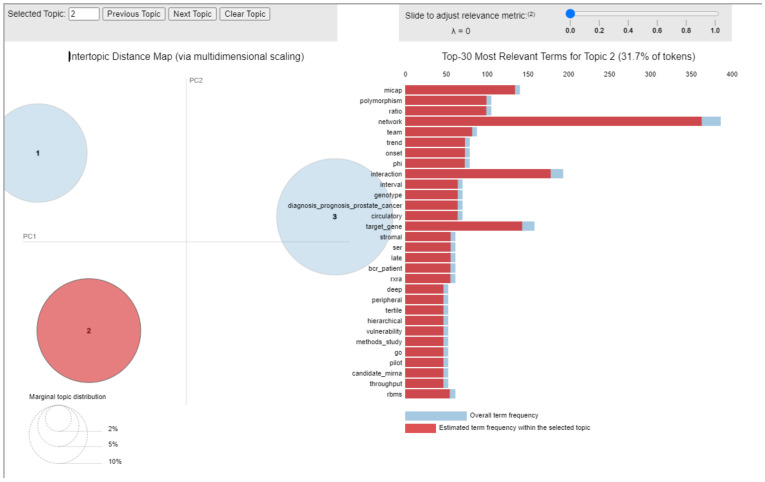
Inter-topic Distance Map related to the topic 2. Circle 1 indicates topic 1, circle 2 is topic 2 and circle 3 is topic 3.

The top-30 most relevant terms of this topic (i.e., the most frequent terms within papers grouped in this topic) (Figure 6), are indicative of a research “network” focus on the evaluation of miRs potential in translation research. Indeed, the 66 analyzed articles aimed to elucidate the relationship of miRs expression with clinicopathological data, and to evaluate the potential of miRs as prognostic and diagnostic biomarkers in PCa.

Because of the molecular heterogeneity of PCa, the ideal biomarker for early diagnosis and prognosis should be capable of identifying potentially aggressive tumors at the stage in which they are still treatable, while minimizing the detection of indolent disease. Aberrant expression of miRs in PCa patients could be a prognostic biomarker, associated with aggressive progression or indicative of poor prognosis. A limitation of these studies is that they often report inconsistent and/or controversial results, due to differences in clinical heterogeneity, study designs and methods of sample collection. Clearly, all these controversial results delay translation from bench to bedside.

Using the MySLR digital platform, we were able to analyze this huge and heterogeneous number of papers, and select and evaluate quality contributions that matched the selected search criteria. In most of the papers, survival was assessed by using the Kaplan–Meier method, differences in survival according to miRs expression were compared by using the log-rank test, while the prognostic values of miRs expression and clinical outcomes were evaluated by Cox regression analysis. Moreover, many analyses were also performed by using bioinformatics tools, such as the “PANTHER” online tool or a deep learning “autoencoder” model.

Therefore, the most relevant publications in the scientific literature reporting miRs as potential prognostic biomarkers in PCa are clustered in this topic (Table 2).

### 3.3. Topic 1—Use of miRs as Biomarkers for PCa in the Clinical Setting

**Figure 7:**
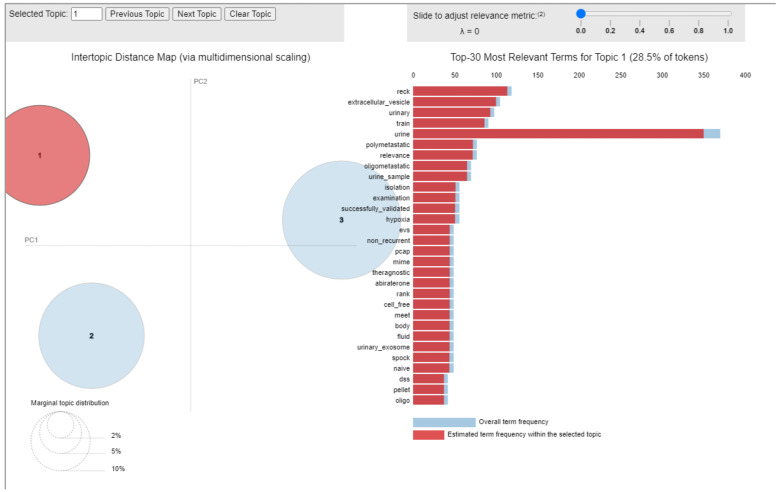
Inter-topic Distance Map related to topic 1. Circle 1 indicates topic 1, circle 2 is topic 2 and circle 3 is topic 3.

As shown in Figure 7, some of the most relevant terms in all 56 papers clustered into topic 1 are “urinary”, “urine”, “urine_sample”. Analysis of all the papers singularly clearly revealed the focus of this topic is the possible use of miRs in the clinical setting as diagnostic or prognostic markers for PCa.

Studies clustered in this topic monitored human miRs in PCa patients by both liquid and tissue biopsies approaches. Scientists assessed miRs expression profiles in PCa tissues and biofluids, including urine, serum/plasma, semen, and prostate secretion fluids at various stages of the disease, and examine their potential as prognostic markers in PCa, as can be seen from Table 3, in which the most relevant papers clustered in the topic are summarized. This research area that investigates circulating miRs as markers is a rapidly developing area; indeed, the topic is based on a growing body of studies whose interest has grown especially over the last 5 years (Figure 4, blue and Table 3). Analysis of miRs in prostate tissue is routinely performed on fresh tissue, but also in formalin fixed paraffin embedded tissue (FFPE) due to the stable nature of miRs, by using microarrays, next generation sequencing (NGS) and qRT-PCR. For many years, biopsies have been the gold standard to determine clinicopathological characteristics of cancer tissues, but the procedure is very aggressive and uncomfortable for patients. During the last years, non-invasive methods have shown relevance, because they could be good indicators for cancer detection at the molecular level. Cancer cells can release miRs, which are stabilized by their incorporation into microvesicles secreted by the prostate, these are detectable in body fluids without requiring invasive biopsies. Several body fluids such as blood/serum, semen, urine, etc. have been used. Detection of miRs in blood/serum has some limitations and is often controversial. Appropriate endogenous controls for miRs quantification in serum are under debate because many mRNA and rRNA species are absent in blood/serum due to circulating RNases. Furthermore, changes in circulating miRs can occur because of therapies, diet, or other factors, thus increasing noise in these assays. However, in addition to serum and plasma, miRs have been identified in other body fluids, in particular semen and urine, which makes them even more interesting biomarkers candidate for PCa. Most of the papers clustered in topic 1 report urine as an excellent option and a reliable non-invasive tool for identifying PCa, and several diagnostic methods have also been described. They are available to detect the presence or absence of miRs involved in the development of the disease by using a non-invasive urine-based test.

## 4. Discussion

Considering the above, advances in early detection are crucial. Scientists have just started to evaluate the role of miRs as clinical biomarkers for PCa detection. Urine-based miR tests seem to be the most useful in PCa diagnosis and prognosis and may help to reduce the number of unnecessary prostate biopsies and guide treatment decisions. Tumor cells release exosomes into biological fluids, and so also into urine, molecules inside are protected from degradation by the exosomal lipid bilayer. As exosomes contain tumor-driven molecules (including miRs), urinary exosomes have been considered ideal substrates for the development of non-invasive biomarkers. Furthermore, the number and composition of exosomal miRs are different between healthy and diseased patients, therefore, the study of novel biomarkers in exosomes is a promising research field for studying PCa prognostic biomarkers [95]. Zhuo et al. investigated whether exosomal miR-141 is an effective biomarker for human PCa. They showed that miR-141 expression was higher in exosomes and in PCa patients than in whole serum and patients with benign prostatic hyperplasia. Expression levels were also significantly higher in metastatic PCa than in localized PCa (*p* < 0.0001) [115]. Recently, levels of specific miRs were also measured in exosomes from urine samples in order to develop a model for the prediction of biochemical recurrence after radical prostatectomy for curative purposes [103]. An alternative method uses field-effect transistor (FET)-based sensors, which allow to measure chemicals and biomolecules with electrical signals. In particular, a label-free urinary miR sensing system was reported, it was based on a disposable and switchable graphene-based electrical sensor with high sensitivity and specificity in urine samples useful as a non-invasive method. This sensor enables rapid and direct detection of target miRs over a wide dynamic range, with a detection limit of up to 10 fM in human urine samples within 20 min, and it also allows for simultaneous quantification of multiple miRs [100]. To identify and validate urinary miRs with the aim of increasing the specificity of PCa diagnosis, several clinicopathological parameters of patients are taken into account. Among these, PSA is used as a clinical biomarker for PCa diagnosis. Guadarrama et al. demonstrated that the model including the miR-100/200b signature significantly outperformed the ability of PSA to discriminate between PCa and benign prostatic hyperplasia [114]. Fredsoe et al. observed expression levels of different miRs by qPCR in cell-free urine samples from patients with benign prostatic hyperplasia and from those with clinically localized PCa. Furthermore, they developed a new diagnostic model of three miRs (miR-222-3p*miR-24-3p/miR-30c-5p) which distinguished benign prostatic hyperplasia from PCa [105]. Moreover, urinary levels of miR-21 also had significant discriminatory power (*p* = 0.010) to separate benign prostatic hyperplasia from PCa by using real time PCR [112]. A different approach is to analyze serial urine samples from patients with localized PCa. Urine miRs validation was generated from three patient cohorts with different Gleason scores. First, temporally stable miRs were measured, and a predictive biomarker of the Gleason score, used as a clinicophatological parameter, was created by using machine-learning techniques [96]. Although most of the papers clustered in topic 1 agree to consider urine as a reliable non-invasive sample for identifying PCa status by testing miRs, the diagnostic methodologies used are several and different, as described above, thus highlighting the limitations of any clinical application of miRs.

Although, as already noted, the research in the field is moving in this direction, before an actual clinical test can be developed further studies are needed, including large sample sets with well-supported validation through long-term clinical data. It seems necessary to establish widely accepted guidelines in the near future, which will determine the best urine-related method, sampling and processing, sample storage, miRs isolation and quantification, quality control and data analysis, in order to minimize the high inter- and intra-tumoral variability. Finally, prior to any clinical application of miRs, optimization is critical to enhance PCa detection, as well as to use miRs in cancer therapy. Moreover, the use of miRs as prognostic markers in PCa may help to define subpopulations of patients with significantly different expected outcomes, who could benefit from different therapies. Patients with a good prognosis may not require additional treatment beyond the primary surgical resection, while patients with a poor prognosis may derive improved survival from adjuvant therapy. Hence, prognostic markers could potentially be “drivers” of cancer progression. Apart from well-noted diagnostic and prognostic values, miRs also provide a potential treatment option for PCa. MiR-based therapy has a great potential to be a more powerful tool in tumor treatment due the simultaneous modulation of multiple genes involved in distinct tumor-related signaling networks. In this view, personalized anticancer therapy is the most ambitious challenge of modern medicine, aiming to identify novel patient-tailored treatments based on the unique features of patient’s disease. This approach, based on miRs delivery, could represent a potential non-toxic successful therapy for a large subset of PCa patients, which could not only decrease the socio-economic costs of this disease, but also improve its burden on patients’ life, thus improving their quality of life.

## 5. Conclusions

Prostate cancer has attracted a great deal of interest due to its high rate of mortality among cancers worldwide [1]. Prostate cancer patients are typically asymptomatic in their early stages, and are often diagnosed too late, thus failing in successful treatment. Although mortality rates have been reduced, thanks to early detection and improved treatment strategies, diagnostic methods are very aggressive for patients and a lot remains to be done to avoid overtreatment.

Extensive studies over the last 15 years have clearly suggested that miRs are critical regulators in PCa progression and development and indicate the use of miRs as promising non-invasive markers of tumoral diagnosis and prognosis. All this huge scientific literature highlights how fascinating but also complicated the world around miRs can be. Studies are often controversial, some questions are being answered, while many new ones need to be answered. Contradictory results between studies can be caused, for example, by differences in the methodology used to analyze and isolate miRs. The use of miRs as markers is promising, but it is not yet a reality in daily clinical practice, and there is still no clear vision of where scientists should turn their attention.

This study aimed to deepen the understanding of existing literature on the role of miRs as potential non-invasive biomarkers in PCa, in terms of major research topics. A machine learning method was used to automatically extract knowledge from scientific literature by means of the LDA algorithm. The innovative concept is based on the ability to independently analyze the texts and identify a certain number of “topics”, and subsequently be able to recognize their presence within the texts themselves. The developed algorithm simulates an intelligence that is as human and complete as possible. Therefore, a systematic review of the literature based on LDA was employed. By analyzing 213 papers we found three main topics that the literature focused on, which are also areas for future research. A first observation that arises quite easily from reading the topics is that the literature passes from the basic research level (topic 3) to the translational level (topic 2), to then consider clinical aspects of increasing complexity (topic 1). Our analysis suggests a prevalence of studies (91) aimed to identify deregulated miRs in PCa, their putative targets and their role in tumoral development and progression. Translational and clinical research studies were a minority (66 and 56 papers, respectively) but their interest is growing more and more over time, thus pointing the direction for future research. Furthermore, our analysis leads us to conclude that several miRs are associated with PCa development and progression, they are indicative of poor prognosis and aggressiveness, are stable under adverse conditions, and can be easily detected in urine. Hence, urinary miRs are valid and promising candidates as non-invasive biomarkers for PCa, as their presence or absence in urine is correlated with that of matched tumor tissues. As highlighted in the Discussion, methodologies used in miRs analysis are several and different, making it necessary to determine the best urine-related method along with accepted guidelines for sampling and processing, quality control and data analysis. To date, we are not yet able to know exactly which miR candidate is the best to be used as a biomarker. Analyzing the papers, we noticed indeed that the number of miRs detected within individuals is different, thus suggesting high variability of miRs within individuals, and high inter- and intra-tumoral variability. Nevertheless, miR-200 family members (including miR-200a, miR-200b, miR-200c, miR-429, and miR-141) were the most repeated miRs in our selected papers, and they could represent potential urine-based biomarkers for PCa detection and prognosis because: (i) their expression is necessary for the maintenance of the epithelial phenotype, as they are important negative regulators of epithelial to mesenchymal transition (EMT), an essential developmental process implicated in cancer metastasis [10,11]; (ii) their expression is deregulated in PCa, in tissue as well as in blood [73]; and (iii) they have unusually high stability in biological fluids, as this is an important prerequisite for usefulness as a biomarker [107,111,112,115]. Of all candidates, miR-141 showed the greatest differential expression (46-fold overexpressed) in PCa patients compared to healthy controls. In this regard, promising results were recently obtained in PCa detection by analyzing miR-141 in urinary exosomes isolated by differential centrifugation [43,73,86,91,108].

Obviously, as already mentioned, further studies and validation in a large tightly defined patient population are needed to confirm the usefulness of these urinary miRs as PCa biomarkers. Although significant efforts remain to be made, we expect this innovative miR-based technology to drastically change medical practice in the foreseeable future.

In conclusion, this is the first time that a text mining technique, led by using an innovative machine learning approach, has been applied to a sample of original scientific articles in a medical setting. The methodology was used to specifically address the role of miRs in PCa, and their potential as non-invasive biomarkers for early diagnosis and prognosis. Certainly, this study could pave the way for other studies with larger cohorts, and it could be applied and extended in order to study other cancers or diseases.

## Figures and Tables

**Table 1:** List of the most relevant and representative papers clustered in the topic 3 over time.

Year	Ref.	miRNAs	Main Regulatory Effect	Clinicopathological Data	Category	Number of Patients	*p* Value
2022	[21]	miR-1273 g-3p	Promotes tumor progression by increasing cell proliferation, migration and invasion	Age	≤65; >65	54; 51	0.378
				PSA (ng/mL)	<10; ≥10	51; 54	0.284
				Differentiation	Poor; well moderate	48; 57	0.139
				Gleason score	≤7; >7	55; 50	0.024
				TNM stage	I-II; III-IV	57; 48	0.008
				Clinical stage	T1- T2	54; 51	0.015
				Lymph node metastasis	No; Yes	56; 49	0.014
2021	[22]	miR-199-5p	Suppresses PCa metastasis by inhibiting EMT pathway	Age	≤65; >65	58; 36	0.424
				PSA (ng/mL)	<10; 10–20; >20	27; 30; 37	0.061
				Gleason score	<7; =7; >7	38;28; 28	<0.001
				Clinical stage	≤T2; ≥T3	65; 29	0.278
				Lymph node involvement	Neg; Pos	89; 5	0.636
				Distal metastases	Yes; No	38; 56	0.003
	[23]	miR-877-5p	Suppresses PCa via forkhead box M1	Age	<60; ≥65	53; 48	0.940
				Tumor size	≤3; >3	62; 39	0.849
				PSA (ng/mL)	≤10; >10	65; 36	0.196
				Surgical margin	Neg; Pos	72; 29	0.433
				Prostate volume (ml)	≤50, >50	66; 35	0.878
				TNM stage	I–II; III	54; 47	0.011
				Gleason score	≤7; >7	68; 33	0.047
	[24]	miR-92b-3p	Suppresses PCa by inhibiting cell proliferation, migration, and invasion	Age	<60; ≥60	41; 67	0.685
				PSA (ng/mL)	<10; ≥10	34; 74	0.009
				Bone metastasis	Neg; Pos	55; 53	0.033
				Gleason score	≤7; >7	59; 49	0.001
2020	[25]	miR-130b	Inhibits PCa angiogenesis via TNF-α/NF-kB/VEGFA axis	N/A	N/A	N/A	N/A
	[26]	miR-495	Promotes cancer progression via KDM5A/miRNA-495/YTHDF2/m6A-MOB3B axis	N/A	N/A	N/A	N/A
	[27]	miR-671-5p	Promotes PCa development and metastasis via NFIA/CRYAB axis	Age	≤72; >72	23; 17	0.31
				Clinical stage	T2; T3–T4	26; 14	0.75
				Gleason score	<7; ≥7	5; 35	0.70
				Lymph node metastasis	N0; N1	28; 12	0.42
				Distant metastasis	M0; M1	25; 15	0.004
	[28]	miR-137- 3p	Inhibits PCa progression via JNK3/EZH2 axis	Gleason score	6; 7; 8	4; 10; 6	N/A
				Grading System	1, 2, 3, 4	6; 4; 4; 6	
				Tumor Stage	I; II; III, IV	4; 9; 6; 1	
	[29]	miR-138-5p	Inhibits PCa progression via FOXC1	Age	<60; ≥60	24; 36	0.830
				Tumor size (cm)	<4; ≥4	28; 32	0.526
				Gleason score	≤7; >7	40; 20	0.025
				Lymph node metastasis	No; Yes	37; 23	0.009
				Bone metastasis	No; Yes	35; 25	0.109
	[30]	miR-140	Inhibits PCa cell invasion and migration via YES proto-oncogene 1	N/A	N/A	N/A	N/A
2019	[31]	miR-515-5p	Inhibits PCa progression via TRIP13	Gleason score	<7; =7; >7	45; 15; 36	<1
				Clinical stage	T1–T2; T3–T4	41; 55	0.007
	[32]	miR-106a-363 cluster	Inhibits PCa progression by inhibiting IFNϒ pathway	N/A	N/A	N/A	N/A
	[33]	miR-148—3p miR-152-3p	Inhibits PCa progression by repressing KLF4	Age	≤65; >65	26; 16	N/A
				PSA (ng/mL)	≤10; >10	23; 19	
				Tumor size (mm)	≤20; >20	20; 22	
				pT-stage	pT2; pT3a; pT3b	19; 16; 7	
				Gleason grade	1; 2; 3; 4; 5	12; 16; 3; 5; 6	
	[34]	miR-214-5p	Inhibits PCa proliferation and migration by increasing levels of CRMP5	N/A	N/A	N/A	N/A
	[35]	miR-455-5p	Suppresses PCa progression by targeting CCR5	Gleason score	<7; =7; >7	32; 55; 19	<0.001
				PSA (ng/mL)	≤10; >10	44; 63	0.006
	[36]	miR-425-5p	Promotes PCa development by targeting FOXJ3	N/A	N/A	N/A	N/A
	[37]	miR-198	Suppresses PCa by targeting MIB1	Gleason score	<7; >7	149; 13	0.02
	[38]	miR-505	Suppresses PCa progression by targeting NRCAM	Tissue	Cancer; non cancer	50; 23	0.432
				Age	≤60; >60	12; 68	0.331
				Gleason score	≤7; >7	31; 19	0.032
				Pathological grade	≤2; >2	4; 46	0.010
				Tumor stage	T1; T2-T4	29; 21	0.351
				Lymph node metastasis	N0; N1	43; 7	15; 2
				Distant metastasis	M0; M1	44; 6	0.093
2018	[39]	miR-373-3p	Inhibits PCa progression by targeting AKT1	N/A	N/A	N/A	N/A
	[40]	miR-1246	Inhibits PCa cell proliferation, invasiveness, and migration via EMT pathway	Pathological stage	pT2a; pT2b; pT3; pT4	3; 17; 11; 36	0.002
				Gleason score	≤7; >7	31; 34	0.7263
				Lymph node metastasis	Yes; No	25; 43	0.0436
				Age	40–59; 60–79	18; 49	0.9576
				Serum PSA	≤6.78; >6.78	32; 33	0.1778
				Race	White; Black	61; 7	0.3528
	[41]	miR-410-3p	Promotes PCa progression by regulating PTEN/AKT/mTOR signaling pathway	Age	<70; ≥70	16; 26	0.596
				Metastasis	No; Yes	9; 33	0.001
				Gleason score	<7; ≥7	19; 23	0.006
				Clinical stage	T1; T2-T3	17; 25	0.003
				PSA levels (ng/mL)	<10; ≥10	20; 22	0.370
	[42]	miR-141	Inhibits PCa cell proliferation, migration, and induces cell apoptosis by targeting RUNX1	N/A	N/A	N/A	N/A
	[43]	miR-29c	Inhibits PCa cell proliferation and glycolysis by inhibiting SLC2A3 expression	Metastasis	No; Yes	127; 14	<0.001
				Gleason score	<7; 8; 9	227; 49; 76	<0.001
				Pathological stage	II; IIIa; IIIb; IV	148; 31; 81; 4	<0.01
2017	[44]	miR-30d	Promotes angiogenesis and tumor growth via MYPT1/c-JUN/VEGFA pathway	Age	<66; ≥66	93; 20	0.613
				PSA levels (ng/mL)	<4; ≥4	22; 89	0.003
				Gleason score	<8; ≥8	87; 19	0.010
				Clinical stage	<T2a; ≥T2a	65; 44	0.007
				Pathological stage	T2a–T2c; T3a–T4	71; 37	0.004
				Metastasis	No; Yes	94; 19	0.001
				Overall survival	Alive; Died	99; 14	0.097
	[45]	miR-30c	Promotes PCa cells invasion by downregulating KRAS protein	N/A	N/A	N/A	N/A
	[46]	miR-2909	Promotes oncogenic functions by attenuating TGFβ signaling	N/A	N/A	N/A	N/A
2016	[47]	miR-24	Inhibits PCa by regulating CDKN1B/p27	Age	≥41; ≤67	N/A	N/A
				PSA levels (ng/mL)	≥4.5; ≤17.7		
				Gleason score	≥5; ≤8		
				TMN	pT2; pT3a		
	[48]	miR-195	Promotes PCa progression by targeting HMGA1	N/A	N/A	N/A	N/A
2015	[49]	miR-503	Suppresses PCa cell proliferation and metastasis by targeting RNF31	Age	<70; ≥70	77; 63	0.886
				Lymph node metastasis	No; yes	124; 16	0.051
				Clinical stage	T1; T2–T3	85; 55	0.004
				Gleason score	<7; =7; >7	65; 34; 41	<0.001
				PSA levels	<4; 4–10; >10	6; 45; 79	<0.001
2014	[50]	miR-224	Inhibits PCa progression by targeting TRIB1	Age	<66; ≥66	89; 25	0.08
				PSA levels (ng/mL)	<4; ≥4	24; 90	0.02
				Gleason score	<8; ≥8;	91; 23	0.09
				Clinical stage	<T2a; ≥T2a	66; 48	0.04
				Pathological stage	T2a; T2c; T3a–T4	76; 38	0.08
				Metastasis	No; Yes	91; 23	<0.001
2013	[51]	miR-4723	Inhibits PCa growth by targeting AbI kinase	Gleason grade	4–6; 7; 8–10	51; 33; 14	N/A
				Pathological stage	pT2; pT3	65; 19	
				Biochemical recurrence	Yes; no	47; 42	
2012	[52]	miR-23b	Suppresses PCa by repressing proto-oncogene Src kinase	Pathological stage	pT2; pT3–pT4	89; 61	<1 × 10^−4^
				Gleason score	4–6; 7; 8–10	46; 52; 34	<0.001
				Biochemical recurrence	yes	36	<1 × 10^−4^
	[53]	miR-708	Promotes PCa progression by regulating CD44+ and AKT2	Age	40–59; 60–89	33; 68	0.6557
				Gleason score	4–6; 7; 8–10	36; 41; 23	8 × 10^−4^
				Pathological stage	pT2; pT3; pT4	57; 42; 2	0.1095
				Biochemical recurrence	Yes; No	24; 76	0.0138
	[5]	miR-205	Inhibits PCa cell migration and metastasis via the EMT pathway	Metastasis	N/A	N/A	<1 × 10^−5^
				Lymph node involvement			<0.01
				Biochemical recurrence			0.00191
				Gleason score			
				PSA levels			

Abbreviations: N/A means Not Applicable.

**Table 2:** List of the most relevant and representative papers clustered in topic 2 over time. N/A: Not Applicable.

Year	Ref.	miRNAs	Bioinformatic Analysis	Clinicopathological Data	Category	Number of Patients	*p* Value
2022	[54]	miR-25-3p, miR-93-3p, miR-122-5p, miR-183-5p, miR-615-3p, miR-7-5p, miR-375 and miR-92a-3p	N/A	Gleason score	6–9	493	<0.01
2021	[55]	miR-146a	N/A	Gleason score	<7; ≥7	100	0.43
				Clinical stage	pT2; pT3		0.004
		mi-100		Biochemical recurrence	Yes; No		0.011
				PSA levels (ng/mL)	<10; ≥10		0.003
	[56]	miR-143, miR-378a	N/A	Overall		494 188; 290; 9 46; 245; 64; 136; 3	5.33 × 10^−9^ <0.001
				TNM stage	2; 3; 4		
				Gleason score	6; 7; 8; 9; 10		
				C-index	0.684		
	[57]	70 miRs	PANTHER	N/A	N/A	N/A	N/A
2020	[58]	miR-17-5p, miR-20a-5p, miR-92a-3p, miR-93-5p	Cancer Genome Atlas	N/A	N/A	N/A	N/A
	[59]	69 miRNAs	CytoHubba	Clinical stage Gleason score PSA levels Race	T2 7 ≤10	49	N/A
2019	[60]	miR-93-5p	Cancer Genome Atlas and Gene Ontology	N/A	N/A	N/A	N/A
	[61]	miR-182	N/A	Age	60	133	
				Race/ethnicity	White, black		0.97
				Gleason score	5–9		0.22
				Clinical stage	T2–T3		0.16
	[62]	miR-142-3p, miR-142-5p, miR-223-3p, miR-342-3p, miR-374b-5p	N/A	PSA levels	4–10 ng/mL	24	0.02
				Gleason score	6–8		
				Clinical stage	T1–T3		
	[63]	miR-21, miR-221	N/A	Gleason score	6–10	100	<0.01
				Clinical stage	T1–T4		
	[64]	miR-21, miR-141, miR-221	N/A	N/A	N/A	N/A	N/A
	[65]	miR-21	Gene Expression Omnibus	N/A	N/A	N/A	N/A
2018	[66]	13 miRNAs	miRcode, Gene Ontology	N/A	N/A	499	<0.05
	[67]	miR-101-3p, miR-145-5p, miR-204-5p, miR-198, miR-152	Gene expression omnibus	N/A	N/A	142	<0.05
	[68]	miR-23a, miR-10b-5p, miR-133a, miR-374-5p	N/A	Age	65	123	
				Clinical stage	T2–T3		
				Gleason score	I–V		
	[69]	miR-15a, miR-16-1	N/A	Age	65	70	0.02, 0.007 0.001
				Gleason score	≤7, >7		
				Clinical stage	≤T2, >T2		
	[70]	miR-21	Cancer genome Atlas; Gene Expression Omnibus	Clinical stage	T2–T4	N/A	<0.001,
				Tumor stage	III–IV		
				PSA levels	≥10		
				Gleason score	≥7		
	[71]	miR-99a-3p	Cancer genome Atlas; Gene Expression Omnibus, Kyoto Encyclopedia of Genes and Genomes	Age	<60, ≥60	201, 203	0.917
				Race	White, black, Asian	146, 7, 2	0.759
				Stage	I–II, III–IV	187, 300	0.347
	[72]	miR-141	N/A	Age	67	30	0.389
				PSA levels (ng/mL)	30		<0.001,
				Prostate volume (g)	89		<0.001,
2017	[73]	miR-200c, miR-200b	N/A	Age	<65, ≥65	30; 72	0.007
				Ancestry	Caucasian; African	79; 23	0.94
				Smoking habit	Yes; No	31; 71	0.96
				Alcohol consumption	Yes; No	58; 44	0.55
				Family history of cancer	Yes; Yes, prostate; No	62; 16; 40	0.09
	[74]	14 miRNAs	N/A	Age	N/A	89	N/A
				PSA levels			
				Metastasis			
	[75]	miR-1	N/A	N/A	N/A	78	<0.001
	[76]	miR-21, miR-34a, miR-125b, miR-126, miR-143, miR-145	N/A	Age	52–71	49	0.016
				PSA level	<10; 10–20; >20	28; 17; 4	
				Gleason score	≤6; 7; ≥8	19; 28; 2	
				Clinical stage	T2a; T2b; T2c; T3a; T3b	4; 5; 32; 3; 5	
	[77]	miR-711	GO, KEGG	Age	<60, ≥60	13; 61	<0.05
				Smoking habits	Yes; No	49; 25	
				PSA levels	Median; High	23; 51	
				Gleason score	7; ≥7	31; 43	
2016	[78]	Let-7c, let-7e, let-7i, miR-26a-5p, miR-26b-5p, miR-24-3p, miR-23b-3p, miR-27-b-3p, miR-106a-5p, miR-20b-5p, miR-18b-5p, miR-19b-2-5p, miR-363-3p, miR-497, miR-195, miR-25-3p, miR-30c-5p, miR-622, miR-874-3p, miR-346, miR-940	N/A	Age	>75	64	N/A
				PSA levels (ng/mL)	>3, <10		
				Gleason score	6; 7; 8	35; 15; 4	
				Clinical stage	cT1c; cT2a; T2b; T2c	40; 4; 8; 12	
	[79]	miR-21	N/A	N/A	N/A	92	<0.05
	[80]	miR-301a	GEO			197	<0.01
	[81]	miR-30c; miR-2013	N/A	Age	<50; >50	21; 23	0.091
				TNM stage	I + II; III + IV	19; 25	0.039
				Metastatic status	Yes; No	18; 26	<0.001
				Clinical stage	T1 + T2; T3; T4	17; 14; 13	0.0167
2015	[82]	miR-1290 miR-375	N/A	Overall		23	N/A
				Gleason score	7; 8; 9	10; 4; 9	
				Clinical stage	T1-T4	23	
	[83]	miR-29a, miR-10a, miR-221	TCGA, GO, KEGG			551	1 × 10^−5^
	[84]	miR-21	N/A	Clinical stage	N/A	75	<0.001
				Lymph node metastasis			
				Tumor differentiation			
2014	[85]	miR-21; miR-141; miR-221	N/A	Age	58.5 ± 7	59	0.0149; <1 × 10^−4^; 2 × 10^−4^
				Race	White		
				Pathologic stage	T2–T3		
				Gleason score	6–8		
	[86]	miR-628-5p	N/A	N/A	N/A	36	<1 × 10^−4^
	[87]	miR-605	N/A	PSA levels (ng/mL)	2–7; 8–10	846	<0.001
				Gleason score			<0.001
				Pathologic stage			<0.001
				Surgical margin			<0.001
	[88]	miR-21	N/A	Age	≤65; <65	357; 176	
				Clinical stage	T2; T3a; T3b	324; 114; 47	<0.001
				PSA levels (ng/mL)	<10; >10	308; 221	<0.001
				Gleason score	6; 7; 8; >8	183; 300; 19; 33	<0.001
				Tumor size (mm)	0–20; >20	250; 285	<0.001
	[89]	miR-7	N/A	N/A	N/A	N/A	0.012;
		miR-221					0.002
		miR-222					0.002
2013	[90]	miR-141, miR-146b-3p, mir-194	N/A	Age	60	16	0.0857
				PSA levels	<10; ≥10	11; 5	0.0282
				Gleason score	7; 9	12; 4	0.001
				Clinical stage	T2-T3	6; 10	0.001
	[91]	miR-224	N/A	Overall	9.23 ± 0.69 64.8 ± 0.74	73	
				PSA levels (ng/mL)			
				Age			
				Gleason score			<0.001
				Clinical stage			0.005
2012	[92]	Let-7e, let-7c, miR-346; miR-622, miR-940, miR-1285	N/A	Age	73 ± 8	105	0.18 <0.001
				PSA levels (ng/mL)	0–4; 4.1–20; >20	42; 28; 31	
				Gleason score	6–7; 8–9	62; 37	
	[93]	miR-96, miR-182, miR-143	GEO	N/A	N/A	N/A	N/A
2011	[94]	miR-16, miR-34a, miR-126, miR-145, miR-205	MicroCosm, KEGG	N/A	N/A	N/A	0.001

**Table 3:** List of the most relevant and representative papers clustered in topic 1 over the time. N/A: Not Applicable.

Year	Ref.	miRNAs	Biological Fluid	Number of Patients	Interval Time of miRNA Processed after Sample Collection
2021	[95]	miR-21, miR-16, miR-142-3p, miR-451, miR-636	Urine	149	Exosomes isolation from samples and incubation overnight at 4 °C
	[96]	miR-3195, let-7b-5p, miR-144-3p, miR-451, miR-148a-3p, miR-512-5p, miR-431-5p	Urine	149	Overnight at −80 °C
	[97]	miR-5100	Plasma	102	After 1 h
	[98]	miR-940	Serum and urine	32	N/A
	[99]	miR4732-3p, mir-98-5p, miR-let-7a-5p, miR-26b-5p, miR-21-5p	Plasma	290	N/A
	[100]	miR-21, miR-1246, miR-let-7b	Urine	10	After 20 min
2020	[101]	miR-182, miR-187	Urine	63	N/A
	[102]	miR-142-3p, miR-142-5p, miR-223-3p	Semen	7	After 30 min at 37 °C
2019	[103]	miR-151 a-5p, miR-204-5p, miR-222-3p, miR-23b-3p, miR-331-3p	Urine	215	Fresh urine samples
	[104]	miR-494	Serum	90	N/A
2018	[105]	miR-222-3p miR-24-3p/miR-30c-5p	Urine	215	N/A
2017	[106]	miR-375, miR-200c-3p, miR-21-5p, let-7a-5p	Plasma	50	Within 2 h
	[107]	miR-21, miR-141, miR-214, miR-375, let-7c	Urine	60	Stored at 4 °C and processed within 4 h
	[108]	miR-193b	Tissue and urine	180	Fresh samples
	[109]	miR-155, miR-152, miR-137 and miR-31	Tissue	129	N/A
	[110]	miR-32-5p, miR-455-4p, miR-184, miR-31-5p, miR-200b-3p, miR-19b-3p, miR-34a-5p, miR-32-5p, miR-143-5p, miR-200b-3p and miR-375	Blood	34	Fresh samples
2016	[111]	miR-200c, miR-605, miR-135a, miR-433 and miR-106a	Serum	16	N/A
	[112]	miR-21, miR-19a and miR-19b	Urine	143	within half an hour
	[113]	miR-410-5p	Serum	149	within 1 h
	[114]	miR-100, miR-200b	Urine		Samples were stored at −80 °C for one week and further processed
2015	[115]	miR-141	Serum	11	1 h at room temperature
	[116]	let-7c, miR-30c, miR-141 and miR-375	Plasma	11	1 h
	[117]	miR-375	Serum	146	Samples stored at −80 °C until RNA isolation
	[118]	miR-133b, miR-221, miR-361-3p	Prostate secretion samples	23	Samples stored at −80 °C until RNA isolation
2014	[119]	miR-187 and miR-182	Tissue and urine	92	Fresh samples
2012	[120]	miR-107 and miR-574-3p	Plasma and urine	78 and 135	10 min plasma samples; stored at 4 °C for up 4 h urine samples
2011	[121]	miR-375 and miR-141	Serum	71	30 min at room temperature
	[122]	384 human miRNAs	Serum	36	N/A
2008	[123]	miR-100, miR-125b, miR-141, miR-143, miR-205, miR296	Plasma and serum	25	Within 2 h

## Data Availability

The data presented in this study are available in this article.

## References

[1] Sung H., Ferlay J., Siegel R.L., Laversanne M., Soerjomataram I., Jemal A., Bray F. (2021). Global Cancer Statistics 2020: GLOBOCAN Estimates of Incidence and Mortality Worldwide for 36 Cancers in 185 Countries. CA Cancer J. Clin..

[2] Gupta S., Coronado G.D., Argenbright K., Brenner A.T., Castaneda S.F., Dominitz J.A., Green B., Issaka R.B., Levin T.R., Reuland D.S. (2020). Mailed fecal immunochemical test outreach for colorectal cancer screening: Summary of a Centers for Disease Control and Prevention-sponsored Summit. CA Cancer J. Clin..

[3] Bartel D.P. (2004). MicroRNAs: Genomics, Biogenesis, Mechanism, and Function. Cell.

[4] Calin G.A., Sevignani C., Dumitru C.D., Hyslop T., Noch E., Yendamuri S., Shimizu M., Rattan S., Bullrich F., Negrini M. (2004). Human microRNA genes are frequently located at fragile sites and genomic regions involved in cancers. Proc. Natl. Acad. Sci. USA.

[5] Tucci P., Agostini M., Grespi F., Markert E.K., Terrinoni A., Vousden K.H., Muller P.A., Dotsch V., Kehrloesser S., Sayan B.S. (2012). Loss of p63 and its microRNA-205 target results in enhanced cell migration and metastasis in prostate cancer. Proc. Natl. Acad. Sci. USA.

[6] Aghdam A.M., Amiri A., Salarinia R., Masoudifar A., Ghasemi F., Mirzaei H. (2019). MicroRNAs as Diagnostic, Prognostic, and Therapeutic Biomarkers in Prostate Cancer. Crit. Rev. Eukaryot. Gene Expr..

[7] Casanova-Salas I., Rubio-Briones J., Fernandez-Serra A., Lopez-Guerrero J.A. (2012). miRNAs as biomarkers in prostate cancer. Clin. Transl. Oncol..

[8] Moher D., Shamseer L., Clarke M., Ghersi D., Liberati A., Petticrew M., Shekelle P., Stewart L.A., Group P.-P. (2015). Preferred reporting items for systematic review and meta-analysis protocols (PRISMA-P) 2015 statement. Syst. Rev..

[9] Gurbuz V., Sozen S., Bilen C.Y., Konac E.J.O.L. (2021). miR-148a, miR-152 and miR-200b promote prostate cancer metastasis by targeting DNMT1 and PTEN expression. Oncol. Lett..

[10] Williams L.V., Veliceasa D., Vinokour E., Volpert O.V.J.P.O. (2013). miR-200b inhibits prostate cancer EMT, growth and metastasis. PLoS ONE.

[11] Basu S., Chaudhary A., Chowdhury P., Karmakar D., Basu K., Karmakar D., Chatterjee J., Sengupta S. (2020). Evaluating the role of hsa-miR-200c in reversing the epithelial to mesenchymal transition in prostate cancer. Gene.

[12] Vulli A., Srinivasu P.N., Sashank M.S.K., Shafi J., Choi J., Ijaz M.F.J.S. (2022). Fine-Tuned DenseNet-169 for Breast Cancer Metastasis Prediction Using FastAI and 1-Cycle Policy. Sensors.

[13] Singh J., Thakur D., Gera T., Shah B., Abuhmed T., Ali F.J.I.A. (2021). Classification and analysis of android malware images using feature fusion technique. IEEE Access.

[14] Denyer D., Tranfield D. (2009). Producing a systematic review. The Sage Handbook of Organizational Research Methods.

[15] Petticrew M. (2001). Systematic reviews from astronomy to zoology: Myths and misconceptions. BMJ.

[16] Cumpston M., Li T., Page M.J., Chandler J., Welch V.A., Higgins J.P., Thomas J. (2019). Updated guidance for trusted systematic reviews: A new edition of the Cochrane Handbook for Systematic Reviews of Interventions. Cochrane Database Syst. Rev..

[17] Ammirato S., Felicetti A., Rogano D., Linzalone R., Corvello V. (2022). Digitalising the Systematic Literature Review process: The MySLR platform. Knowl. Manag. Res. Pract..

[18] Blei D.M., Ng A.Y., Jordan M.I. (2003). Latent dirichlet allocation. J. Mach. Learn. Res..

[19] Moher D., Liberati A., Tetzlaff J., Altman D.G., Group P. (2009). Preferred reporting items for systematic reviews and meta-analyses: The PRISMA statement. PLoS Med..

[20] Chen Z., Liu B. Topic Modeling using Topics from Many Domains, Lifelong Learning and Big Data. Proceedings of the 31st International Conference on Machine Learning, Proceedings of Machine Learning Research.

[21] Chang Y., Deng Q., Guan Z., Cheng Y., Sun Y. (2022). MiR-1273 g-3p Promotes Malignant Progression and has Prognostic Implications in Prostate Cancer. Mol. Biotechnol..

[22] Zhao Z., Zhao S., Luo L., Xiang Q., Zhu Z., Wang J., Liu Y., Luo J. (2021). miR-199b-5p-DDR1-ERK signalling axis suppresses prostate cancer metastasis via inhibiting epithelial-mesenchymal transition. Br. J. Cancer.

[23] Yang B., Diao H., Wang P., Guan F., Liu H. (2021). microRNA-877-5p exerts tumor-suppressive functions in prostate cancer through repressing transcription of forkhead box M1. Bioengineered.

[24] Wang G., Cheng B., Jia R., Tan B., Liu W. (2021). Altered expression of microRNA-92b-3p predicts survival outcomes of patients with prostate cancer and functions as an oncogene in tumor progression. Oncol. Lett..

[25] Mu H.Q., He Y.H., Wang S.B., Yang S., Wang Y.J., Nan C.J., Bao Y.F., Xie Q.P., Chen Y.H. (2020). MiR-130b/TNF-alpha/NF-kappaB/VEGFA loop inhibits prostate cancer angiogenesis. Clin. Transl. Oncol..

[26] Du C., Lv C., Feng Y., Yu S. (2020). Activation of the KDM5A/miRNA-495/YTHDF2/m6A-MOB3B axis facilitates prostate cancer progression. J. Exp. Clin. Cancer Res..

[27] Zhu Z., Luo L., Xiang Q., Wang J., Liu Y., Deng Y., Zhao Z. (2020). MiRNA-671-5p Promotes prostate cancer development and metastasis by targeting NFIA/CRYAB axis. Cell Death Dis..

[28] Zang Y., Zhu J., Li Q., Tu J., Li X., Hu R., Yang D. (2020). miR-137-3p Modulates the Progression of Prostate Cancer by Regulating the JNK3/EZH2 Axis. Onco. Targets Ther..

[29] Zhang D., Liu X., Zhang Q., Chen X. (2020). miR-138-5p inhibits the malignant progression of prostate cancer by targeting FOXC1. Cancer Cell Int..

[30] Zhao S., Jie C., Xu P., Diao Y. (2020). MicroRNA-140 inhibit prostate cancer cell invasion and migration by targeting YES proto-oncogene 1. J. Cell Biochem..

[31] Zhang X., Zhou J., Xue D., Li Z., Liu Y., Dong L. (2019). MiR-515-5p acts as a tumor suppressor via targeting TRIP13 in prostate cancer. Int. J. Biol. Macromol..

[32] Lo U.G., Pong R.C., Yang D., Gandee L., Hernandez E., Dang A., Lin C.J., Santoyo J., Ma S., Sonavane R. (2019). IFNgamma-Induced IFIT5 Promotes Epithelial-to-Mesenchymal Transition in Prostate Cancer via miRNA Processing. Cancer Res..

[33] Feng F., Liu H., Chen A., Xia Q., Zhao Y., Jin X., Huang J. (2019). miR-148-3p and miR-152-3p synergistically regulate prostate cancer progression via repressing KLF4. J. Cell Biochem..

[34] Zheng C., Guo K., Chen B., Wen Y., Xu Y. (2019). miR-214-5p inhibits human prostate cancer proliferation and migration through regulating CRMP5. Cancer Biomark..

[35] Xing Q., Xie H., Zhu B., Sun Z., Huang Y. (2019). MiR-455-5p Suppresses the Progression of Prostate Cancer by Targeting CCR5. Biomed. Res. Int..

[36] Zhang J.Y., Su X.P., Li Y.N., Guo Y.H. (2019). MicroRNA-425-5p promotes the development of prostate cancer via targeting forkhead box J3. Eur. Rev. Med. Pharmacol. Sci..

[37] Ray J., Hoey C., Huang X., Jeon J., Taeb S., Downes M.R., Boutros P.C., Liu S.K. (2019). MicroRNA198 suppresses prostate tumorigenesis by targeting MIB1. Oncol. Rep..

[38] Ling X.H., Fu H., Chen Z.Y., Lu J.M., Zhuo Y.J., Chen J.H., Zhong W.D., Jia Z. (2019). miR505 suppresses prostate cancer progression by targeting NRCAM. Oncol. Rep..

[39] Qu H.W., Jin Y., Cui Z.L., Jin X.B. (2018). MicroRNA-373-3p inhibits prostate cancer progression by targeting AKT1. Eur. Rev. Med. Pharmacol. Sci..

[40] Bhagirath D., Yang T.L., Bucay N., Sekhon K., Majid S., Shahryari V., Dahiya R., Tanaka Y., Saini S. (2018). microRNA-1246 Is an Exosomal Biomarker for Aggressive Prostate Cancer. Cancer Res..

[41] Zhang Y., Zhang D., Lv J., Wang S., Zhang Q. (2018). miR-410-3p promotes prostate cancer progression via regulating PTEN/AKT/mTOR signaling pathway. Biochem. Biophys Res. Commun..

[42] Xu S., Ge J., Zhang Z., Zhou W. (2018). miR-141 inhibits prostatic cancer cell proliferation and migration, and induces cell apoptosis via targeting of RUNX1. Oncol. Rep..

[43] Li J., Fu F., Wan X., Huang S., Wu D., Li Y. (2018). Up-regulated miR-29c inhibits cell proliferation and glycolysis by inhibiting SLC2A3 expression in prostate cancer. Gene.

[44] Lin Z.Y., Chen G., Zhang Y.Q., He H.C., Liang Y.X., Ye J.H., Liang Y.K., Mo R.J., Lu J.M., Zhuo Y.J. (2017). MicroRNA-30d promotes angiogenesis and tumor growth via MYPT1/c-JUN/VEGFA pathway and predicts aggressive outcome in prostate cancer. Mol. Cancer.

[45] Zhang J., Wang X., Wang Y., Peng R., Lin Z., Wang Y., Hu B., Wang J., Shi G. (2017). Low expression of microRNA-30c promotes prostate cancer cells invasion involved in downregulation of KRAS protein. Oncol. Lett..

[46] Ayub S.G., Kaul D., Ayub T. (2017). An androgen-regulated miR-2909 modulates TGFbeta signalling through AR/miR-2909 axis in prostate cancer. Gene.

[47] Lynch S.M., McKenna M.M., Walsh C.P., McKenna D.J. (2016). miR-24 regulates CDKN1B/p27 expression in prostate cancer. Prostate.

[48] Zhang X., Tao T., Liu C., Guan H., Huang Y., Xu B., Chen M. (2016). Downregulation of miR-195 promotes prostate cancer progression by targeting HMGA1. Oncol. Rep..

[49] Guo J., Liu X., Wang M. (2015). miR-503 suppresses tumor cell proliferation and metastasis by directly targeting RNF31 in prostate cancer. Biochem. Biophys Res. Commun..

[50] Lin Z.Y., Huang Y.Q., Zhang Y.Q., Han Z.D., He H.C., Ling X.H., Fu X., Dai Q.S., Cai C., Chen J.H. (2014). MicroRNA-224 inhibits progression of human prostate cancer by downregulating TRIB1. Int. J. Cancer.

[51] Arora S., Saini S., Fukuhara S., Majid S., Shahryari V., Yamamura S., Chiyomaru T., Deng G., Tanaka Y., Dahiya R. (2013). MicroRNA-4723 Inhibits Prostate Cancer Growth through Inactivation of the Abelson Family of Nonreceptor Protein Tyrosine Kinases. PLoS ONE.

[52] Majid S., Dar A.A., Saini S., Arora S., Shahryari V., Zaman M.S., Chang I., Yamamura S., Tanaka Y., Deng G. (2012). miR-23b represses proto-oncogene Src kinase and functions as methylation-silenced tumor suppressor with diagnostic and prognostic significance in prostate cancer. Cancer Res..

[53] Saini S., Majid S., Shahryari V., Arora S., Yamamura S., Chang I., Zaman M.S., Deng G., Tanaka Y., Dahiya R. (2012). miRNA-708 control of CD44(+) prostate cancer-initiating cells. Cancer Res..

[54] Santo G.D., Frasca M., Bertoli G., Castiglioni I., Cava C. (2022). Identification of key miRNAs in prostate cancer progression based on miRNA-mRNA network construction. Comput. Struct. Biotechnol. J..

[55] Camargo J.A., Lopes R.E., Ferreira G.F.D., Viana N.I., Guimaraes V., Leite K.R.M., Nahas W.C., Srougi M., Antunes A.A., Reis S.T. (2021). The role of single nucleotide polymorphisms of miRNAs 100 and 146a as prognostic factors for prostate cancer. Int. J. Biol. Markers.

[56] Wang T.H., Lee C.Y., Lee T.Y., Huang H.D., Hsu J.B., Chang T.H. (2021). Biomarker Identification through Multiomics Data Analysis of Prostate Cancer Prognostication Using a Deep Learning Model and Similarity Network Fusion. Cancers.

[57] Liu H., Li L., Fan Y., Lu Y., Zhu C., Xia W. (2021). Construction of Potential Gene Expression and Regulation Networks in Prostate Cancer Using Bioinformatics Tools. Oxid. Med. Cell Longev..

[58] Wei J., Yin Y., Deng Q., Zhou J., Wang Y., Yin G., Yang J., Tang Y. (2020). Integrative Analysis of MicroRNA and Gene Interactions for Revealing Candidate Signatures in Prostate Cancer. Front. Genet..

[59] Parra-Medina R., Lopez-Kleine L., Ramirez-Clavijo S., Payan-Gomez C. (2020). Identification of candidate miRNAs in early-onset and late-onset prostate cancer by network analysis. Sci. Rep..

[60] Yang Y., Jia B., Zhao X., Wang Y., Ye W. (2019). miR-93-5p may be an important oncogene in prostate cancer by bioinformatics analysis. J. Cell Biochem..

[61] Baumann B., Acosta A.M., Richards Z., Deaton R., Sapatynska A., Murphy A., Kajdacsy-Balla A., Gann P.H., Nonn L. (2019). Association of High miR-182 Levels with Low-Risk Prostate Cancer. Am. J. Pathol..

[62] Barcelo M., Castells M., Bassas L., Vigues F., Larriba S. (2019). Semen miRNAs Contained in Exosomes as Non-Invasive Biomarkers for Prostate Cancer Diagnosis. Sci. Rep..

[63] Ibrahim N.H., Abdellateif M.S., Thabet G., Kassem S.H., El-Salam M.A., El-Leithy A.A., Selim M.M. (2019). Combining PHI and miRNAs as Biomarkers in Prostate Cancer Diagnosis and Prognosis. Clin. Lab..

[64] Marrone M.T., Joshu C.E., Peskoe S.B., De Marzo A.M., Heaphy C.M., Lupold S.E., Meeker A.K., Platz E.A. (2019). Adding the Team into T1 Translational Research: A Case Study of Multidisciplinary Team Science in the Evaluation of Biomarkers of Prostate Cancer Risk and Prognosis. Clin. Chem..

[65] Xue Z., Xi Q., Liu H., Guo X., Zhang J., Zhang Z., Li Y., Yang G., Zhou D., Yang H. (2019). miR-21 promotes NLRP3 inflammasome activation to mediate pyroptosis and endotoxic shock. Cell Death Dis..

[66] Xu N., Wu Y.P., Yin H.B., Xue X.Y., Gou X. (2018). Molecular network-based identification of competing endogenous RNAs and mRNA signatures that predict survival in prostate cancer. J. Transl. Med..

[67] Lin Y., Chen F., Shen L., Tang X., Du C., Sun Z., Ding H., Chen J., Shen B. (2018). Biomarker microRNAs for prostate cancer metastasis: Screened with a network vulnerability analysis model. J. Transl. Med..

[68] Schmidt L., Fredsoe J., Kristensen H., Strand S.H., Rasmussen A., Hoyer S., Borre M., Mouritzen P., Orntoft T., Sorensen K.D. (2018). Training and validation of a novel 4-miRNA ratio model (MiCaP) for prediction of postoperative outcome in prostate cancer patients. Ann. Oncol..

[69] Zidan H.E., Abdul-Maksoud R.S., Elsayed W.S.H., Desoky E.A.M. (2018). Diagnostic and prognostic value of serum miR-15a and miR-16-1 expression among egyptian patients with prostate cancer. IUBMB Life.

[70] Chen Z., Zhan Y., Chi J., Guo S., Zhong X., He A., Zheng J., Gong Y., Li X., Zhou L. (2018). Using microRNAs as Novel Predictors of Urologic Cancer Survival: An Integrated Analysis. EBioMedicine.

[71] Yan H.B., Zhang Y., Cen J.M., Wang X., Gan B.L., Huang J.C., Li J.Y., Song Q.H., Li S.H., Chen G. (2018). Expression of microRNA-99a-3p in Prostate Cancer Based on Bioinformatics Data and Meta-Analysis of a Literature Review of 965 Cases. Med. Sci. Monit..

[72] Ali R., El Tabbakh S., El Delgawy W., Kotb A., Desouky M.N. (2018). microRNA-141 as a diagnostic and prognostic biomarker for prostate cancer in Egyptian population: Pilot study. Afr. J. Urol..

[73] Souza M.F., Kuasne H., Barros-Filho M.C., Ciliao H.L., Marchi F.A., Fuganti P.E., Paschoal A.R., Rogatto S.R., Colus I.M.S. (2017). Circulating mRNAs and miRNAs as candidate markers for the diagnosis and prognosis of prostate cancer. PLoS ONE.

[74] Lin H.M., Mahon K.L., Spielman C., Gurney H., Mallesara G., Stockler M.R., Bastick P., Briscoe K., Marx G., Swarbrick A. (2017). Phase 2 study of circulating microRNA biomarkers in castration-resistant prostate cancer. Br. J. Cancer.

[75] Wei W., Leng J., Shao H., Wang W. (2017). MiR-1, a Potential Predictive Biomarker for Recurrence in Prostate Cancer After Radical Prostatectomy. Am. J. Med. Sci..

[76] Zedan A.H., Blavnsfeldt S.G., Hansen T.F., Nielsen B.S., Marcussen N., Pleckaitis M., Osther P.J.S., Sorensen F.B. (2017). Heterogeneity of miRNA expression in localized prostate cancer with clinicopathological correlations. PLoS ONE.

[77] Waseem M., Ahmad M.K., Srivatava V.K., Rastogi N., Serajuddin M., Kumar S., Mishra D.P., Sankhwar S.N., Mahdi A.A. (2017). Evaluation of miR-711 as Novel Biomarker in Prostate Cancer Progression. Asian Pac. J. Cancer Prev..

[78] Cochetti G., Poli G., Guelfi G., Boni A., Egidi M.G., Mearini E. (2016). Different levels of serum microRNAs in prostate cancer and benign prostatic hyperplasia: Evaluation of potential diagnostic and prognostic role. Onco Targets Ther..

[79] Yang B., Liu Z., Ning H., Zhang K., Pan D., Ding K., Huang W., Kang X.L., Wang Y., Chen X. (2016). MicroRNA-21 in peripheral blood mononuclear cells as a novel biomarker in the diagnosis and prognosis of prostate cancer. Cancer Biomark..

[80] Sun Y., Jia X., Hou L., Liu X. (2016). Screening of Differently Expressed miRNA and mRNA in Prostate Cancer by Integrated Analysis of Transcription Data. Urology.

[81] Huang Z., Zhang L., Yi X., Yu X. (2016). Diagnostic and prognostic values of tissue hsa-miR-30c and hsa-miR-203 in prostate carcinoma. Tumour Biol..

[82] Huang X., Yuan T., Liang M., Du M., Xia S., Dittmar R., Wang D., See W., Costello B.A., Quevedo F. (2015). Exosomal miR-1290 and miR-375 as prognostic markers in castration-resistant prostate cancer. Eur. Urol..

[83] Xiaoli Z., Yawei W., Lianna L., Haifeng L., Hui Z. (2015). Screening of Target Genes and Regulatory Function of miRNAs as Prognostic Indicators for Prostate Cancer. Med. Sci. Monit..

[84] Huang W., Kang X.L., Cen S., Wang Y., Chen X. (2015). High-Level Expression of microRNA-21 in Peripheral Blood Mononuclear Cells Is a Diagnostic and Prognostic Marker in Prostate Cancer. Genet. Test Mol. Biomark..

[85] Zheng Q., Peskoe S.B., Ribas J., Rafiqi F., Kudrolli T., Meeker A.K., De Marzo A.M., Platz E.A., Lupold S.E. (2014). Investigation of miR-21, miR-141, and miR-221 expression levels in prostate adenocarcinoma for associated risk of recurrence after radical prostatectomy. Prostate.

[86] Srivastava A., Goldberger H., Dimtchev A., Marian C., Soldin O., Li X., Collins S.P., Suy S., Kumar D. (2014). Circulatory miR-628-5p is downregulated in prostate cancer patients. Tumour Biol..

[87] Huang S.P., Levesque E., Guillemette C., Yu C.C., Huang C.Y., Lin V.C., Chung I.C., Chen L.C., Laverdiere I., Lacombe L. (2014). Genetic variants in microRNAs and microRNA target sites predict biochemical recurrence after radical prostatectomy in localized prostate cancer. Int. J. Cancer.

[88] Melbo-Jorgensen C., Ness N., Andersen S., Valkov A., Donnem T., Al-Saad S., Kiselev Y., Berg T., Nordby Y., Bremnes R.M. (2014). Stromal expression of MiR-21 predicts biochemical failure in prostate cancer patients with Gleason score 6. PLoS ONE.

[89] Santos J.I., Teixeira A.L., Dias F., Maurício J., Lobo F., Morais A., Medeiros R. (2014). Influence of peripheral whole-blood microRNA-7 and microRNA-221 high expression levels on the acquisition of castration-resistant prostate cancer: Evidences from in vitro and in vivo studies. Tumor Biol..

[90] Selth L.A., Townley S.L., Bert A.G., Stricker P.D., Sutherland P.D., Horvath L.G., Goodall G.J., Butler L.M., Tilley W.D. (2013). Circulating microRNAs predict biochemical recurrence in prostate cancer patients. Br. J. Cancer.

[91] Mavridis K., Stravodimos K., Scorilas A. (2013). Downregulation and prognostic performance of microRNA 224 expression in prostate cancer. Clin. Chem..

[92] Chen Z.H., Zhang G.L., Li H.R., Luo J.D., Li Z.X., Chen G.M., Yang J. (2012). A panel of five circulating microRNAs as potential biomarkers for prostate cancer. Prostate.

[93] Alshalalfa M., Bader G.D., Goldenberg A., Morris Q., Alhajj R. (2012). Detecting microRNAs of high influence on protein functional interaction networks: A prostate cancer case study. BMC Syst. Biol..

[94] Watahiki A., Wang Y., Morris J., Dennis K., O’Dwyer H.M., Gleave M., Gout P.W., Wang Y. (2011). MicroRNAs associated with metastatic prostate cancer. PLoS ONE.

[95] Shin S., Park Y.H., Jung S.H., Jang S.H., Kim M.Y., Lee J.Y., Chung Y.J. (2021). Urinary exosome microRNA signatures as a noninvasive prognostic biomarker for prostate cancer. NPJ Genom. Med..

[96] Jeon J., Olkhov-Mitsel E., Xie H., Yao C.Q., Zhao F., Jahangiri S., Cuizon C., Scarcello S., Jeyapala R., Watson J.D. (2020). Temporal Stability and Prognostic Biomarker Potential of the Prostate Cancer Urine miRNA Transcriptome. J. Natl. Cancer Inst..

[97] Mello-Grand M., Bruno A., Sacchetto L., Cristoni S., Gregnanin I., Dematteis A., Zitella A., Gontero P., Peraldo-Neia C., Ricotta R. (2021). Two Novel Ceramide-Like Molecules and miR-5100 Levels as Biomarkers Improve Prediction of Prostate Cancer in Gray-Zone PSA. Front. Oncol..

[98] Rajendiran S., Maji S., Haddad A., Lotan Y., Nandy R.R., Vishwanatha J.K., Chaudhary P. (2021). MicroRNA-940 as a Potential Serum Biomarker for Prostate Cancer. Front. Oncol..

[99] Giglio S., De Nunzio C., Cirombella R., Stoppacciaro A., Faruq O., Volinia S., Baldassarre G., Tubaro A., Ishii H., Croce C.M. (2021). A preliminary study of micro-RNAs as minimally invasive biomarkers for the diagnosis of prostate cancer patients. J. Exp. Clin. Cancer Res..

[100] Kim S., Park S., Cho Y.S., Kim Y., Tae J.H., No T.I., Shim J.S., Jeong Y., Kang S.H., Lee K.H. (2021). Electrical Cartridge Sensor Enables Reliable and Direct Identification of MicroRNAs in Urine of Patients. ACS Sens..

[101] Nayak B., Khan N., Garg H., Rustagi Y., Singh P., Seth A., Dinda A.K., Kaushal S. (2020). Role of miRNA-182 and miRNA-187 as potential biomarkers in prostate cancer and its correlation with the staging of prostate cancer. Int. Braz. J. Urol..

[102] Mercadal M., Herrero C., Lopez-Rodrigo O., Castells M., de la Fuente A., Vigues F., Bassas L., Larriba S. (2020). Impact of Extracellular Vesicle Isolation Methods on Downstream Mirna Analysis in Semen: A Comparative Study. Int. J. Mol. Sci..

[103] Fredsoe J., Rasmussen A.K.I., Mouritzen P., Borre M., Orntoft T., Sorensen K.D. (2019). A five-microRNA model (pCaP) for predicting prostate cancer aggressiveness using cell-free urine. Int. J. Cancer.

[104] Cai B., Peng J.H. (2019). Increased Expression of miR-494 in Serum of Patients with Prostate Cancer and its Potential Diagnostic Value. Clin. Lab..

[105] Fredsoe J., Rasmussen A.K.I., Thomsen A.R., Mouritzen P., Hoyer S., Borre M., Orntoft T.F., Sorensen K.D. (2018). Diagnostic and Prognostic MicroRNA Biomarkers for Prostate Cancer in Cell-free Urine. Eur. Urol. Focus.

[106] Endzelins E., Berger A., Melne V., Bajo-Santos C., Sobolevska K., Abols A., Rodriguez M., Santare D., Rudnickiha A., Lietuvietis V. (2017). Detection of circulating miRNAs: Comparative analysis of extracellular vesicle-incorporated miRNAs and cell-free miRNAs in whole plasma of prostate cancer patients. BMC Cancer.

[107] Foj L., Ferrer F., Serra M., Arevalo A., Gavagnach M., Gimenez N., Filella X. (2017). Exosomal and Non-Exosomal Urinary miRNAs in Prostate Cancer Detection and Prognosis. Prostate.

[108] Torres-Ferreira J., Ramalho-Carvalho J., Gomez A., Menezes F.D., Freitas R., Oliveira J., Antunes L., Bento M.J., Esteller M., Henrique R. (2017). MiR-193b promoter methylation accurately detects prostate cancer in urine sediments and miR-34b/c or miR-129-2 promoter methylation define subsets of clinically aggressive tumors. Mol. Cancer.

[109] Daniunaite K., Dubikaityte M., Gibas P., Bakavicius A., Rimantas Lazutka J., Ulys A., Jankevicius F., Jarmalaite S. (2017). Clinical significance of miRNA host gene promoter methylation in prostate cancer. Hum. Mol. Genet..

[110] Rodríguez-Báez A., Comoto-Santacruz D.A., Huerta-Núñez L.F.E., Campos-Saucedo J.G., Estrada-Carrasco C.E.J. (2017). Circulating miRNA expression as a clinical option for the detection of prostate cancer. Rev. Mex. Urol..

[111] Alhasan A.H., Scott A.W., Wu J.J., Feng G., Meeks J.J., Thaxton C.S., Mirkin C.A. (2016). Circulating microRNA signature for the diagnosis of very high-risk prostate cancer. Proc. Natl. Acad. Sci. USA.

[112] Stuopelyte K., Daniunaite K., Jankevicius F., Jarmalaite S. (2016). Detection of miRNAs in urine of prostate cancer patients. Medicina.

[113] Wang J., Ye H., Zhang D., Hu Y., Yu X., Wang L., Zuo C., Yu Y., Xu G., Liu S. (2016). MicroRNA-410-5p as a potential serum biomarker for the diagnosis of prostate cancer. Cancer Cell Int..

[114] Salido-Guadarrama A.I., Morales-Montor J.G., Rangel-Escareno C., Langley E., Peralta-Zaragoza O., Cruz Colin J.L., Rodriguez-Dorantes M. (2016). Urinary microRNA-based signature improves accuracy of detection of clinically relevant prostate cancer within the prostate-specific antigen grey zone. Mol. Med. Rep..

[115] Li Z., Ma Y.Y., Wang J., Zeng X.F., Li R., Kang W., Hao X.K. (2016). Exosomal microRNA-141 is upregulated in the serum of prostate cancer patients. Onco Targets Ther..

[116] Kachakova D., Mitkova A., Popov E., Popov I., Vlahova A., Dikov T., Christova S., Mitev V., Slavov C., Kaneva R. (2015). Combinations of serum prostate-specific antigen and plasma expression levels of let-7c, miR-30c, miR-141, and miR-375 as potential better diagnostic biomarkers for prostate cancer. DNA Cell Biol..

[117] Wach S., Al-Janabi O., Weigelt K., Fischer K., Greither T., Marcou M., Theil G., Nolte E., Holzhausen H.J., Stohr R. (2015). The combined serum levels of miR-375 and urokinase plasminogen activator receptor are suggested as diagnostic and prognostic biomarkers in prostate cancer. Int. J. Cancer.

[118] Guzel E., Karatas O.F., Semercioz A., Ekici S., Aykan S., Yentur S., Creighton C.J., Ittmann M., Ozen M. (2015). Identification of microRNAs differentially expressed in prostatic secretions of patients with prostate cancer. Int. J. Cancer.

[119] Casanova-Salas I., Rubio-Briones J., Calatrava A., Mancarella C., Masia E., Casanova J., Fernandez-Serra A., Rubio L., Ramirez-Backhaus M., Arminan A. (2014). Identification of miR-187 and miR-182 as biomarkers of early diagnosis and prognosis in patients with prostate cancer treated with radical prostatectomy. J. Urol..

[120] Bryant R.J., Pawlowski T., Catto J.W., Marsden G., Vessella R.L., Rhees B., Kuslich C., Visakorpi T., Hamdy F.C. (2012). Changes in circulating microRNA levels associated with prostate cancer. Br. J. Cancer.

[121] Brase J.C., Johannes M., Schlomm T., Falth M., Haese A., Steuber T., Beissbarth T., Kuner R., Sultmann H. (2011). Circulating miRNAs are correlated with tumor progression in prostate cancer. Int. J. Cancer.

[122] Moltzahn F., Olshen A.B., Baehner L., Peek A., Fong L., Stoppler H., Simko J., Hilton J.F., Carroll P., Blelloch R. (2011). Microfluidic-based multiplex qRT-PCR identifies diagnostic and prognostic microRNA signatures in the sera of prostate cancer patients. Cancer Res..

[123] Mitchell P.S., Parkin R.K., Kroh E.M., Fritz B.R., Wyman S.K., Pogosova-Agadjanyan E.L., Peterson A., Noteboom J., O’Briant K.C., Allen A.J. (2008). Circulating microRNAs as stable blood-based markers for cancer detection. Proc. Natl. Acad. Sci. USA.

